# Neurological symptoms among dental assistants: a cross-sectional study

**DOI:** 10.1186/1745-6673-3-10

**Published:** 2008-05-18

**Authors:** BE Moen, BE Hollund, T Riise

**Affiliations:** 1 Institute of Public Health and Primary Health Care, University of Bergen, Kalfarveien 31, N-5018 Bergen, Norway; 2 Department of occupational medicine, Haukeland University Hospital, N-5009 Bergen, Norway

## Abstract

**Background:**

Dental assistants help the dentist in preparing material for filling teeth. Amalgam was the filling material mostly commonly used in Norway before 1980, and declined to about 5% of all fillings in 2005. Amalgam is usually an alloy of silver, copper, tin and mercury. Copper amalgam, giving particularly high exposure to mercury was used in Norway until 1994. Metallic mercury is neurotoxic. Few studies of the health of dental assistants exist, despite their exposure to mercury. There are questions about the existence of possible chronic neurological symptoms today within this working group, due to this exposure. The aim of this study was to compare the occurrence of neurological symptoms among dental assistants likely to be exposed to mercury from work with dental filling material, compared to similar health personnel with no such exposure.

**Methods:**

All dental assistants still at work and born before 1970 registered in the archives of a trade union in Hordaland county of Norway were invited to participate (response rate 68%, n = 41), as well as a similar number of randomly selected assistant nurses (response rate 87%, n = 64) in the same age group. The participants completed a self-administered, mailed questionnaire, with questions about demographic variables, life-style factors, musculoskeletal, neurological and psychosomatic symptoms (Euroquest).

**Results:**

The dental assistants reported significant higher occurrence of neurological symptoms; psychosomatic symptoms, problems with memory, concentration, fatigue and sleep disturbance, but not for mood. This was found by analyses of variance, adjusting for age, education, alcohol consumption, smoking and personality traits. For each specific neurological symptom, adjusted logistic regression analyses were performed, showing that these symptoms were mainly from arms, hands, legs and balance organs.

**Conclusion:**

There is a possibility that the higher occurrence of neurological symptoms among the dental assistants may be related to their previous work exposure to mercury amalgam fillings. This should be studied further to assess the clinical importance of the reported symptoms.

## Background

Mercury is known to be a potential health hazard, both for kidneys, the nervous system and reproduction [1-3]. Persons employed in the dental profession might have been exposed to metallic mercury during their work with the dental filling material amalgam. Amalgam has been the main dental filling material in Norway from 1945 to the mid-1980s. One of the amalgam filling materials used was the alloy copper amalgam [4]. It was prepared by heating a tablet, and this could give concentrations of mercury fumes in the air above 1.0 mg/m^3^, 20 times the limit value in Norway at the time [5]. Other types of amalgam alloys and different preparation methods have also been applied, causing less mercury exposure. In 1981 the Norwegian Health Authorities recommended the dentists to avoid the use of copper amalgam, due to possible adverse health effects. This request was repeated in 1994, as the compound was still in use. After this last request, the use of copper amalgam declined to almost zero.

Preparing dental fillings is one of the main work tasks for dental assistants, although dentists some times perform this work as well. Several studies have confirmed the mercury exposure in dental offices [6-9]. Mercury vapour levels in dental clinics have been shown to exceed the limit values in Canada in 1983 [10]. Little effort has been made to differentiate between the mercury exposure of dentists and dental assistants, but it seems like the dental assistants may have higher exposure levels [11].

As dental assistants may have been exposed to high levels of mercury during their work with amalgam, and especially during work with copper amalgam, this might have caused chronic adverse effects in their nervous system. However, few studies have examined such effects among dental assistants exposed to mercury, and the studies are small and show inconsistent results [11,12]. Recent studies indicate a relation between urine mercury levels and neurological symptoms and adverse results on neuropsychological tests of motor function among dental assistants and dentists [13,14]. However, these studies analyse data mostly related to current exposure conditions, but also suggest that previous exposure might be of importance for present symptoms[14]. This supports the hypothesis that past exposure among these types of personnel might cause chronic neurological symptoms.

There are several challenges related to examination of symptoms from the nervous system. Several such self-administered questionnaires have been elaborated [15,16], but they all have weaknesses. The EUROQUEST questionnaire was designed in 1992 by the European Neurotoxic Solvents Toxicity network (EURONEST), for use in epidemiological studies on neurotoxicity [17]. The questionnaire has been evaluated and found to have a high internal consistency on assessed domains [18]. It has a specific advantage by including questions on personality, making adjustment for personality possible. Due to these different advantages of this questionnaire, it was chosen for the present study.

The hypothesis of the present study was that the occurrence of neurotoxic symptoms, especially neurological symptoms is higher among dental assistants likely to be exposed to past high levels of mercury during work with dental filling material, compared to health personnel with no occupational mercury exposure. The information might be useful in medical examinations of dental assistants.

## Methods

### Study design and subjects

A cross-sectional study among dental assistants was performed in Hordaland County, Norway in 2005. One out of two trade unions for dental assistants in Norway had a good member archive. This union provided a list of all dental assistants who were members of the union and still at work (67 years or younger). All dental assistants from this list, born before 1970, were invited to participate (n = 60). The age restriction made likely for a least some of the participants to have worked with copper amalgam, as this filling material was reduced in Norway after 1994. The union also provided a similar list of assistant nurses, an occupational group with no known mercury exposure at work, and 75 of these were randomly selected for the study. Two had moved outside the country, thus 73 were invited to participate. The assistant nurses were chosen as a reference group as they normally have about the same educational level as the dental assistants. They work at hospitals or similar institutions and care for patients under medical treatment, and are not in dental offices.

### Questionnaire

The participants were mailed a questionnaire and a letter telling that this was a pilot study of work and health among health personnel. Dental filling material was not mentioned in the information. The participants were asked to fill in the questionnaire and to return it to the University in a prestamped envelope within two weeks.

The questionnaire enquired about age, current working position, number of years working as a dental assistant or assistant nurse, previous diseases, alcohol consumption (units per month), present smoking (yes/no) and number of cigarettes smoked. In addition, the standardized questionnaire EUROQUEST was included [17,19,20]. The questionnaire was translated from English to Norwegian and back again, using standard procedures for translations. The EUROQUEST questionnaire measures neurological symptoms (11 items), psychosomatic symptoms (15 items), mood (11 items), memory (6 items), concentration (4 items), fatigue (7 items), sleep disturbances (5 items). In addition the EUROQUEST includes questions on anxiety (6 items) and perception of health status and life (4 items), usually used for adjusting for personality traits [20]. Each item was scored from 1 to 4 according to its frequency. The domains acute symptoms and environmental susceptibility from the original EUROQUEST were not included, as these questions focused on exposures, which we wanted to avoid in the present study to reduce information bias. Scores were calculated for neurological symptoms, psychosomatic symptoms, mood, memory, concentration, fatigue, sleep disturbances and anxiety/general health by summarizing the scores from each group of questions. In addition, the questionnaire included items concerning musculoskeletal symptoms, using a modification of the Standardized Nordic Questionnaire [21,22] The questions were phrased: "Have you at any time the last 12 months, had ache, pain or discomfort in (body area)?" The body areas were head, neck, shoulders, elbows, hands, upper back, lower back, hips, knees and ankle/feet. The answers were given on a five-point scale. These questions were added after a suggestion from the trade union involved, as they were particularly interested in the topic. As the occurrence of musculoskeletal symptoms might be high among health personnel [23,24], it was likely that the workers would be motivated to answer the questionnaire when this subject was included, although the topic was not any main aim of the study.

### Statistical Analysis

Differences among the dental assistants and the assistant nurses in age, years at work in question, alcohol units and number of cigarettes smoked daily were analyzed by t-tests. Differences in education and smoking status were tested using chi-square tests. Differences in mean scores of the EUROQUEST scales between the dental assistants and the reference group were examined by analyses of variance, adjusting for age, education, alcohol consumption (units per week), smoking (cigarettes per week), years at work as well as for personality traits (anxiety and general health). The adjusted mean differences were also expressed as effect size by dividing the difference by the standard deviation in the total study population. Each neurological symptom was also categorized (0/1) into seldom/never and sometimes/often/very often, and the occurrence of each symptom was compared between the groups by logistic regression analyses, adjusting for age (below or above 55 years), education (primary school/collage or university), alcohol consumption (below or above 4 units per month), current smoking (yes/no) and personality traits (score categories below or above mean for both groups). The chosen cut points for age and alcohol consumption were the mean levels for the whole population. Logistic regression analyses with similar adjustments were also performed for the different musculoskeletal symptoms. Due to a correlation between age and years at work (Pearson's correlation coefficient = 0.53, p < 0.000), only age was included in the main analyses. However, additional analyses were also performed including the years at work. Adjusted odds ratios and 95% confidence intervals were calculated.

### Ethics

The Regional Committee for Medical Research Ethics in Health Region West of Norway, and The Norwegian Social Science Data Services (NSD) approved the project.

## Results

The response rate was 68% for dental assistants and 87% for the assistant nurses. The groups had similar educational levels (Table 1). The dental assistants were older, had more years at work, and higher alcohol consumption than the assistant nurses. More assistant nurses were smoking. The dental assistants had started their career in this occupation between 1956 and 1994, and fifty percent of them had been working more than 30 years in this work (Table 1). All except two had started this work before 1980. The groups did not differ regarding previous diseases, except for one dental assistant who had had a brain tumour. This person was excluded from the further analyses.

**Table 1:** Characteristics of the study population

	**Dental assistants (n = 41) **	**Assistant nurses (n = 64)**	**p-value**
	**Mean (SD) range**	**Mean (SD) range**	
**Age**	57.8 (6.7) 37–67	52.5 (8.3) 36–67	0.001*
**Years at this particular work**	29.7 (8.2) 11–42	17.5 (11.8) 1–40	0.001*
**Alcohol units in a month**	4.8 (3.8) 0–15	2.6 (3.0) 0–12	0.010*
**Cigarettes smoked daily**	3.7 (5.6) 0–20	7.2 (6.3) 0–20	0.004*

	**No (percent)**	**No (percent)**	

**Educational level**			
**- Only primary school**	32 (79)	45 (70)	0.079**
**- Primary school and college**	9 (21)	16 (25)	
**- University**	0	3 (5)	
**Present smokers**	7 (17)	32 (50)	0.001**
**Years at this particular work**			
**-<10**	0	20 (34)	
**11–20**	8 (18)	17 (26)	
**21–30**	13 (32)	18 (27)	
**>30**	20 (50)	7(13)	

* Student's t-test** Chi-square test

Regarding musculoskeletal symptoms, no differences were found between the groups in a logistic regression analysis, adjusting for age, education, alcohol consumption and current smoking. This was also the case when the variable years at work was included in the model. The dental assistants reported markedly and significantly higher occurrence of neurological symptoms, psychosomatic symptoms, memory, concentration, fatigue and sleep disturbance than the reference group. This was found when adjusting for age, education level and lifestyle factors, as well as personality traits (Table 2). The largest difference (effect size) was found for memory deficit. The groups did not differ significantly concerning mood, anxiety and perception of health status and life. The last two factors were still included in the analyses of variance as covariates to adjust for personality traits. Including years at work in the analysis in addition to age did not change the results.

**Table 2:** Differences between dental assistants (n = 40) and assistant nurses (n = 64) in scores on domains of the questionnaire EUROQUEST, tested by univariate analyses of variance

**Domain**	**Unadjusted data**		**Adjusted data**		
	**Dental assistants **	**Assistant nurses**	**p-value**	Effect size*	**R**^2^
	**Mean/SD**	**Mean/SD**			

**Neurological symptoms**	18.1/5.0	14.6/4.0	0.028	0.49	0.367
**Psychosomatic symptoms**	21.6/5.9	18.8/4.0	0.024	0.50	0.347
**Mood**	18.8/5.9	16.7/4.8	0.257	0.24	0.410
**Memory deficit**	11.4/4.1	9.1/2.6	0.003	0.59	0.401
**Concentration difficulties**	6.7/1.9	5.6/1.6	0.045	0.50	0.182
**Fatigue**	14.7/5.7	11.8/4.3	0.023	0.49	0.389
**Sleep disturbance**	10.1/3.3	7.9/2.4	0.010	0.52	0.338

^1^Adjusted for age, education, alcohol consumption (units per week), smoking (cigarettes per week) and personality traits (anxiety and general health).

^2^Effect size= The difference between the mean values of the two occupational groups, divided by the standard deviation.

SD= standard deviation. R^2 ^= Adjusted R squared

All single neurological symptoms recorded, except "felt slow in carrying out your daily activities", occurred more in the group of dental assistants (Table 3). Further, seven out of eleven symptoms were significantly more frequent. Including years at work in the analysis in addition to age did not change the results.

**Table 3:** Neurological symptoms among 40 dental assistants with probable previous exposure to mercury during work, compared with 64 assistant nurses

**Neurological symptom**	**Dental assistants (%)**	**Assistant nurses (%)**	OR (95%CI)*
**Unintentionally dropping things**	60	19	9.0 (2.5–32.3)
**Felt weak in your arms and legs**	78	55	3.0 (1.0–9.2)
**Felt less in your arms and legs**	60	38	2.1 (0.8–5.7)
**Felt numbness or heaviness in your arms and legs**	66	56	2.6 (0.9–7.8)
**Felt tingling in your arms and legs**	64	34	4.0 (1.3–12.0)
**Had problems with balance**	53	13	7.7 (2.2–27.1)
**Noticed changes in sense of smell or taste**	29	6	8.6 (1.7–43.2)
**Noticed less sensation in your face**	17	2	8.5 (0.9–85.6)
**Had difficulty controlling hand movements**	39	8	5.5 (1.2–20.5)
**Felt slow in carrying out your daily activities**	32	38	0.8 (0.3–2.6)
**Felt shakiness in your hands**	44	16	6.8 (1.9–24.6)

*OR (95%CI) = Odds ratio with 95 percent confidence interval, calculated by logistic regression analyses, adjusting for age, education, alcohol consumption, smoking and personality traits (anxiety and general health).

## Discussion

The dental assistants reported markedly and significantly more neurological symptoms, psychosomatic symptoms, memory loss, concentration difficulties, fatigue and sleep disturbance than a reference group of assistant nurses. The memory loss seemed to be most important.

The possible exposure to mercury among the dental assistants during their work with filling material might be an explanatory factor for this finding, as this exposure was not likely among the referents. However, we had no specific information about the individual exposure to mercury during the work among these women, as the questionnaire contained no such questions to reduce recall bias. The dental assistants in the present study had been working at a time when copper amalgam was being used in Norway, and they were very likely to have been exposed to mercury in their dental work. This is supported by a study of mercury in urine samples among dental assistants and dentists in Hordaland performed in 1985. The study demonstrates similar exposure levels for mercury in this group compared to Norwegian dental personnel elsewhere in the country [25].

Other neurotoxins may be in use in dental offices, but as there is no documentation of this exposure, these assumptions are speculative. For instance, in several countries, the neurotoxin nitrous oxide has been used extensively in dental offices as an anaesthetic agent. However, this gas has in general been used relatively little in Norwegian dental offices.

The central nervous system has been described to be vulnerable for chronic exposure to low levels of inorganic mercury over several years [26]. Tremor, nervousness and memory disturbances have also been reported [27-29]. Previous studies of dental personnel are few. One study of neuropsychological functioning [12] has shown reduced short-term memory among dental auxiliaries. Two Scottish studies have shown high prevalence of memory disturbance among dentists [30,31], and similar findings among dental personnel have been shown in Sweden [11].

Psychomotor tests of dental personnel have shown a relation between mercury exposure and lower scores on the Intentional Hand Steadiness Test and finger tapping, although the general mercury levels were quite low [13,32]. The neurological symptoms we found clearly show that the dental assistants have problems with the hands and arms, and this is consistent with the studies mentioned. However, dentistry requires controlled hand movements and precision. Symptoms from arms and hands may be noticed earlier among dental workers than in other occupational groups, as mentioned in a Swedish study of musculoskeletal symptoms among dental personnel [33]. However, this Swedish study also included dentists, who handle vibrating hand tools, and this might have given symptoms from arms and hands as well, and the population is not quite comparable. Nevertheless, early notification of symptoms can probably not alone explain the reported neurological symptoms in the present study, as symptoms independent of the hands were reported as well. Also, there was no difference between the groups regarding questions about musculoskeletal pain in the extremities.

The dental assistants in the present study did not have any different mood than the reference group. This differs from other studies [34,35]. However, the differences found might be due to our adjustment for personality traits, as our unadjusted data showed reduced mood among the dental assistants compared to the reference group.

We used EUROQUEST, which has been designed to explore various neuropsychiatric symptoms caused by neurotoxic agents in occupational epidemiological studies [17,20]. EUROQUEST seems to be particularly valid for memory symptoms [17,18]. The largest difference between the groups in our study was found for memory symptoms.

The response rate was different in the two groups, as the assistant nurses had a higher response rate than the dental assistants. This might be caused by the inclusion of questions about musculoskeletal symptoms, as this is a subject often debated among assistant nurses in Norway, giving them a higher motivation for answering the questionnaire. However, we do not know this for sure, and there might be other causes for the different response rate as well. This may influence the validity of the comparison of these two occupational groups, and must be remembered when interpreting the results.

There were several differences among the two groups. The dental assistants were older and had been in their profession longer. This is probably caused by the fact that assistant nurses are a relatively new profession in Norway, and many women have started this education and work in adult age. Age may be associated with higher prevalence of the symptoms studied here. Therefore adjustment for age was performed in this study. In addition, we adjusted for smoking, alcohol consumption and education, as there were differences in these factors as well. Both groups had very low alcohol consumption, compared to Norwegian standards [36], but the alcohol consumption was higher among the dental assistants. Alcohol consumption and smoking habits often correlate, but this was not the case in the present study. Smoking was highest among the assistant nurses, while quite few of the dental assistants did smoke. However, it has previously been shown that assistant nurses have a high prevalence of smoking in Norway [37], and it has been suggested that this is related to the social climate at work. There are no such studies among dental assistants, but they have a different working situation than the nurses, as they work in small offices and not in large hospital units. This work difference might explain the different smoking habits. Due to the differences found between the groups, we performed a rather conservative adjustment procedure in the analyses. However, the groups still differed significantly in the symptom scores, despite the low number of participants. The differences were large, with an effect size around 0.5 for several of the scales.

With the cross-sectional design, there is a possibility of a healthy worker effect, implying that the problems might be even higher within this occupational group than shown here. The participants were still at work in this study, meaning that their clinical impairment due to the reported symptoms was therefore low.

## Conclusion

The study indicates that dental assistants with a probable past exposure to mercury from work with amalgam have higher occurrence of neurological symptoms than a control group of workers who had not been exposed to mercury. As we had no objective assessment of exposure, the results must be interpreted with caution concerning mercury as a causative factor. The results of this study, indicate the need of further studies of dental assistants, to evaluate the possibility of previous mercury exposure as a causative agent to nervous system symptoms and clinical neurological disease among such workers today.

## Competing interests

The authors declare that they have no competing interests.

## Authors' contributions

BEM, TR and BEH planned the study, BEH and BEM gathered the data, BEM and TR analysed the data and BEM, TR and BEH participated in the discussion of the results and the writing of the manuscript, although BEM was in charge of the writing process. All authors read and approved the final manuscript.
